# A Turbulent Cloud, a Viral Menace

**DOI:** 10.3201/eid2911.AC2911

**Published:** 2023-11

**Authors:** Byron Breedlove

**Affiliations:** Centers for Disease Control and Prevention, Atlanta, Georgia, USA

**Keywords:** art science connection, emerging infectious diseases, art and medicine, about the cover, public health, Ralph Steadman, Viral Menace, A Turbulent Cloud, A Viral Menace, respiratory infections, SARS-CoV-2, COVID-19, viruses, influenza, influenza virus, respiratory syncytial virus, vaccinations, pandemic

**Figure:**
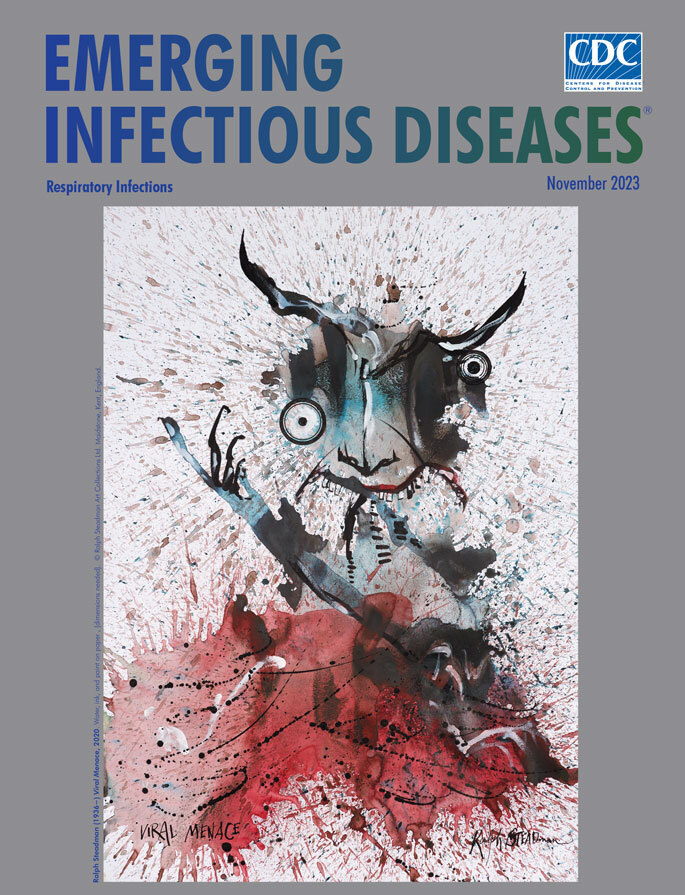
**Ralph Steadman (1936−) *Viral Menace*, 2020.** Water, ink, and paint on paper, 35.25 in x 24.5 in/89.5 cm x 62.23 cm. © Ralph Steadman Art Collections Ltd. Maidstone, Kent, England.

A passage from the biographical information on the Ralph Steadman Art Collection website for the British artist and illustrator is revealing. “One of Ralph’s favourite pastimes growing up was to make model aeroplanes. He would rush home from school and would always complete any outstanding homework before allowing himself to indulge in his hobby. This work ethic has remained with him and later, when he began working with Hunter S. Thompson, would lead to no end of irritation to his tall, trans-Atlantic friend when Ralph would often have completed his drawings before Hunter had written a word.”

After publication of their best known collaboration, *Fear and Loathing in Las Vegas* (first published as a two-part article in Rolling Stone magazine in 1971 and then as a book in 1972), Steadman and Thompson (who was an American journalist and writer who became a counterculture icon) were together catapulted into the popular zeitgeist. A short biographical note on the Tate Museum website states that Steadman “is a British illustrator best known for his collaboration with the American writer Hunter S. Thompson.” 

However, Steadman’s voluminous portfolio reveals a restless creativity extending well beyond his illustrations linked to Thompson’s “gonzo journalism.” Steadman has created political cartoons for various magazines; scathing caricatures of politicians; British postal stamps; product labels; artwork for album, CD, and DVD covers; and a pair of books on what he calls “boids” (one depicting extinct birds and the other endangered ones). He also illustrated many other books, including a 1967 edition of *Alice in Wonderland* and an out-of-print (in English) 1995 edition of *Animal Farm*. 

Steadman himself is the subject of the 2012 documentary *For No Good Reason*. In her review for National Public Radio, the late film and art critic Pat Dowell wrote, “Steadman’s drawings are a ferocious tangle of ink blotches and lines that famously distort but also reveal their subjects.” His most recent book, *Ralph Steadman: A Life in Ink*, is a curated retrospective that spans much of his career. Steadman’s website describes the 300-page book as “a pandemic project,” which was started in November 2019, just before the world learned about the emergence of COVID-19, and which was completed in the fall of 2020 via the online conferencing platforms and file-sharing technologies that provided lifelines for so many people during sustained lockdowns and isolation. 

*Viral Menace*, featured on this month’s cover, comes from a small group of works labeled as Steadman’s “Lockdown Portfolio” (S. Williams, Ralph Steadman Art Collection, pers. comm., 2023 Jan 23), In a review of Steadman’s book from the *Guardian,* journalist Nadja Sayej wrote, “It’s the very last image in the book that sums up 2020. It’s a drawing called *Viral Menace*, a portrait of COVID-19. It looks like an ink-splatted demon over a sea of blood. A walking nightmare, if there ever was one.”

This drawing of a so-called walking nightmare could be seen as a somewhat realistic, if unintentional, depiction of how respiratory viruses, such as SARS-CoV-2, are spread through coughing and sneezing. In a 2021 article about how airborne pathogens are transmitted, researchers Linsey C. Marr and Julian W. Tang describe “the transfer of pathogens in respiratory fluid from 1 person to another” via respiratory droplets of various sizes “emitted as part of a turbulent cloud.” In Steadman’s drawing, the viewer is confronted by a distorted, twisted visage, barely recognizable as human, with eyes askew and an arm extended. This central shape floats in a miasmic turbulent cloud, and the malevolent red mist dominates the lower portion of the image.

Steadman achieves this splattered effect through his “dirty water technique,” as described by Sayej and others, which involves flinging the leftover water used to clean his paintbrushes onto a clean sheet of paper and letting it dry. He then revisits the spattered paper, which at that point looks like a first pass by Jackson Pollock or an image for a Rorschach test, but it provides a jumping off point for the artist. According to Dowell, Steadman remarked, “You don’t pencil in anything; you just start going and see where it leads you." 

Steadman’s drawing was an artistic reaction to the COVID-19 pandemic in 2020. The world is in a different place in 2023, but respiratory viruses, such as SARS-CoV-2, influenza virus, and respiratory syncytial virus, still circulate globally, causing substantial morbidity and mortality. Some populations, including older people, young children, pregnant women, and people with underlying health conditions, are more vulnerable to severe disease from those three respiratory viruses. Key public health actions to help mitigate the combined impact of those current viral menaces include vaccinations that protect against COVID-19, influenza, and respiratory syncytial virus, combined with proven nonpharmaceutical measures such as handwashing, wearing masks, and improving indoor ventilation..
